# The role of HIF in angiogenesis, lymphangiogenesis, and tumor microenvironment in urological cancers

**DOI:** 10.1007/s11033-023-08931-2

**Published:** 2023-12-12

**Authors:** Shen Lin, Yueyang Chai, Xiangyi Zheng, Xin Xu

**Affiliations:** 1 The First Affiliated Hospital, Zhejiang University School of Medicine, Hangzhou, Zhejiang China; 2 The Second Affiliated Hospital, Zhejiang University School of Medicine, Hangzhou, Zhejiang China

**Keywords:** HIF, Angiogenesis, Lymphangiogenesis, Tumor microenvironment, Urological cancers

## Abstract

Typically associated with solid tumors, hypoxia contributes to tumor angiogenesis and lymphangiogenesis through various molecular mechanisms. Accumulating studies indicate that hypoxia-inducible factor is the key transcription factor coordinating endothelial cells to respond to hypoxia in urological cancers, mainly renal cell carcinoma, prostate cancer, and bladder cancer. Moreover, it has been suggested that tumor hypoxia in tumor microenvironment simultaneously recruits stromal cells to suppress immune activities. This review summarizes the mechanisms by which HIF regulates tumorigenesis and elaborates on the associations between HIF and angiogenesis, lymphangiogenesis, and tumor microenvironment in urological cancers.

## Introduction

Urological tumors, mainly renal cell carcinoma (RCC), prostate cancer (PCa) and bladder cancer (BCa), make up 13.1% of newly diagnosed cases and account for 7.9% of all cancer-related deaths [1]. Urological tumors are solid tumors in which hypoxia is generated due to their rapid growth and inadequate nutrient supply. Hypoxia-inducible factors (HIFs) regulate various biological processes induced by hypoxia [2]. Angiogenesis and lymphangiogenesis are two major processes that depend on HIFs to respond to hypoxia. Through these processes, new vessels are formed from pre-existing vessels, and these vessels communicate with stromal components of tumor microenvironment (TME), promoting immunosuppression. This review presents an overview of HIF-dependent angiogenesis, lymphangiogenesis, and the association between such processes with tumor microenvironment in urologic cancers and discusses novel applications and challenges of targeted therapy development for clinical translation.

## HIF activity in urologic cancers

### Regulation of HIF

HIFs, including HIF-1, HIF-2, and HIF-3, constitute a group of transcription factors mediating hypoxia-related responses. HIF-1 was first discovered through an analysis of the human EPO gene. It was later confirmed that HIF-1 consists of two elements: HIF-1α and HIF-1β [3].

HIFs are heterodimers with two subunits, and the stimulation of HIF signalling pathways requires the assembly of alpha units (HIF1α, HIF2α, and HIF3α) and beta units (HIF1β/ARNT1, ARNT2, and ARNT3) [4]. The homeostasis of HIFs is regulated by a family of hydroxylases and the hydroxylation reaction depends on oxygen.

Under normoxic conditions, prolyl hydroxylases are enzymes that facilitate the hydroxylation of HIF-1/2α, and then the ubiquitin ligase complex comprising Von Hippel–Lindau (VHL) recognizes HIF-1/2α hydroxylated proline residues, triggering the ubiquitination of HIF-1/2α [5]. Finally, the 26 S proteasome degraded HIF-1/2α. The hydroxylation of asparagine residues by Factor Inhibiting HIF-1 prevents HIF from engaging with p300 and CREB-binding protein, thereby inhibiting HIF-1/2α transactivation [6]. A hypoxic environment inhibits hydroxylation reactions, resulting in the accumulation of HIF-1/2α. When two subunits of HIF-1/2 bind to cis-acting elements of target genes, a complex is formed, which promotes transcription of the target genes [3].

HIF-1α and HIF-2α target different gene transcripts. HIF-1α can upregulate the glycolytic enzymes expression, while HIF-2α participates in iron metabolism [7]. HIF-3α is an isoform of HIF-α that has been relatively less studied; however, the role of HIF-3α is known to be distinct from that of HIF-α. Inhibitory PAS domain protein, a spliced variant of HIF-3α, negatively regulates HIF-1α, whereas HIF-2α activates the HIF signalling pathway [8].

### RCC

In clear cell RCC (ccRCC) patients, deletions or mutations of the VHL gene are connected with high levels of HIF expression, and elevated HIF levels are linked with poor outcomes [9]. On chromosome 3, the VHL gene is located at cytoband 3p25-26. One mutant allele of VHL is present in individuals with VHL disease, and tumorigenesis is largely dependent on the simultaneous inactivation of both alleles of VHL [10]. Recently, the somatic biallelic deactivation of VHL has been a common occurrence in most sporadic ccRCC. Somatic mutations of VHL have been reported at varying rates, with studies describing frequencies as high as 91% [11].

As part of the ubiquitin ligase complex, the VHL gene product (pVHL), is indispensable for HIF signaling. Loss of pVHL leads to VHL failure to recognize HIF-α subunits. Therefore, the stability of HIF-α is increased, consequently enhancing the transcriptional activities of the HIF target genes under normoxic conditions.

HIF-1 and HIF-2 appear to play distinct roles in ccRCC. ccRCCs that exclusively express HIF-2 exhibit a higher degree of malignancy compared to ccRCCs expressing both HIF-1 and HIF-2 [12]. Phenotype assays showed that HIF-1α functions as a tumor suppressor and HIF-2α functions as a tumor-promoting factor. HIF-1α suppresses the expression of proto-oncogenes, but HIF-2α upregulates oncogenic genes [13]. This is in contrast to the majority of tumors, where HIF-1 and HIF-2 are commonly considered oncogenes. In this particular scenario, there could potentially be an interplay between HIF-1 and HIF-2 contributing to this phenomenon.

### BCa

In primary BCa samples, HIF-1α has been observed via nuclear HIF-1α immunostaining in a vast majority of tumor cells. No HIF-1α expression has been observed in normal tissue [14]. HIF-1α positively correlates with histological grade of tumors, VEGFR levels, and microvessel density (MVD). BCa patients with HIF-1α overexpression showed poor survival outcomes [14]. The heightened expression of HIF-1α induced by hypoxia stimulates the transcription of downstream genes, regulating activities like angiogenesis, nutrient metabolism, and cell cycle progression [15]. HIF-1 and HIF-2 exhibit a synergistic effect in bladder cancer. HIF-2 has been observed to exhibit staining within the stromal region of tumors, and its heightened expression has been correlated with inferior histological grades [16].

### PCa

HIF-1α is elevated in both PCa samples and human PCa cell lines [17]. Upregulated HIF-1α has been reported to be correlated to increased biochemical recurrence, metastasis, and castration resistance in PCa [18, 19]. PCa patients with elevated HIF-2 expression also have poor disease-specific survival [20].

HIF is intricately regulated through genetic and epigenetic mechanisms in PCa. The gene amplification that has been detected by comparative genomic hybridization in PC3 cell lines may partially explain the overexpression of HIF-1α [17]. Single nucleotide polymorphisms (SNPs) may contribute to the upregulation of certain genes. Two SNPs, C1772T and G1790A, identified in castration-resistant PCa (CRPC), may result in the collection of the HIF-1α protein [21].

In general, both HIF-1 and HIF-2 play similar roles in promoting tumor growth in prostate cancer and bladder cancer. However, in renal tumors, HIF-1 and HIF-2 exhibit mutual antagonism, suggesting a regulatory relationship between them and resulting in differential effects on downstream target genes and pathways.

## HIF and angiogenesis

Compact neoplasms initially lack vasculature and rely on oxygen diffused from host vessels. The proliferation or hypertrophy of tumor cells leads to elevated oxygen consumption by tumor cells, which culminates in diminished oxygen accessibility, causing a reduction in hydroxylase function and enhanced HIF transcription. Angiogenesis is an adjustable reaction to hypoxia, and it is commandeered by malignant cells to induce their own oxygen supply and sustain neoplasm expansion. HIFs function as important angiogenic gene regulators. Activation of HIF in tumor and stromal cells leads to an escalation in mRNA level of vascular endothelial growth factor (VEGF) [2, 22]. Other genes involved in angiogenesis regulated by HIF include placental growth factor, platelet-derived growth factor (PDGF), stromal-derived factor, stem cell factor and angiopoietin 2. These factors interact with receptors on the cell membrane, activating specific signalling pathways that lead to the formation of new blood vessels, promoting tumor progression [2, 22]. Hypoxia-induced effects mainly influence two cell types, endothelial cells (ECs), the endothelial layer of blood vessels, and pericytes, which encircle blood vessels. During angiogenesis, endothelial cells respond to VEGF by initiating new blood vessel sprouting. ECs then differentiate into specialized vascular endothelial cells to enhance vessel formation. They became hypoxic because new vessels are located far from well-perfused blood vessels. The hypoxic microenvironment results in metabolic shift involving elevated glycolysis in ECs, and increasingly, fatty acid oxidation in biological activities [23]. ECs serve as barriers preventing the invasion of malignant cells, and the hypoxia modulators HIF-2 and HIF-1 generate opposite implications on barrier function in an iNOS-dependent manner [24]. Pericytes, which form a layer on the exterior of endothelial cells, control the contractility and permeability of blood vessels. PDGF impairs the adhesion of pericytes to blood vessels during neovascularization [25].

Angiogenesis is a complex biological process, and the VEGF family plays a dominant role in regulating angiogenesis. In VEGF family, VEGF-A is the most angiogenic relevant component, and it interacts with VEGFR-2 to impact neovascularization [26].

### Angiogenesis in RCC

Heterogeneity is a characteristic of RCC and can be categorized into three histopathologic types: chromophobe RCC (chRCC), papillary RCC (pRCC), and ccRCC. Type 1 pRCC is more likely to arise from familial mutations of c-Met, whereas Type 2 pRCC is correlated to mutations in fumarate hydratase. Those suffering from Birt–Hogg–Dubé syndrome are prone to developing chRCCs. In most ccRCCs, both alleles of the VHL gene are inactivated because of mutations, chromosomal aberrations, or VHL promoter hypermethylation [9]. Mutations in TCEB1, encoding the elongin C module involved in ubiquitination, have been recently identified in ccRCCs [11]. The loss of pVHL and elongin C function leads to the accumulation of HIF-1α plus HIF-2α.

In normal tubular cells, only the HIF-1α isoform has been detected via immunohistochemical approach [9, 27]. Both HIF-1α as well as HIF-2α have been found in precancerous lesions of VHL-deficient kidney disease patients. In ccRCC tumors, around 30% of patients do not express HIF-1, and its expression is inversely correlated with tumor malignancy. Survival analysis also indicates a favorable prognosis associated with HIF-1 expression [9, 28, 29]. HIF-1 and HIF-2 reciprocally inhibit each other at the epigenetic and protein levels, indicating a regulatory relationship between them [13, 30]. Hoefflin et al. demonstrated that HIF-1α exerted an indispensable effect on the formation of VHL mutant ccRCC mouse model, and deletion of HIF-2α exerted little impact on tumor onset [31].

VEGF binds to VEGFR and promotes angiogenesis. In ccRCC, loss of pVHL correlates with increased expression of VEGF, which results in higher TNM stages and worse prognosis in patients. HIF-2α knockdown alleviated VEGF expression in 786-O VHL-deficient cells, supporting an angiogenic role for VEGF in renal tumors [27]. In addition, in HIF-1α deficient cells, targeting HIF-2 can regulate the expression of VEGF [32].

These results suggested that the two HIF isoforms facilitate cancer progression in different oncogenic periods and HIF-2 plays a central role in angiogenesis. Presently, targeted therapies for RCC predominantly focus on HIF-2, such as Belzutifan and PT2399. However, targeting the interaction between HIF-1 and HIF-2 can be a promising approach. When combined with drugs targeting HIF-2, it may enhance the efficacy of anti-angiogenic treatment.

### Angiogenesis in BCa

The isoforms of HIF-α seemed to exert merged effects to accelerate the vascularization and progression of BCa. HIF-1α was correlated with VEGF and MVD in BCa [33]. HIF-2α was discovered in the stroma area around tumors, especially in the regions surrounding necrotic tumors, and was positively associated with VEGF expression [16]. Mesenchymal cells secrete cytokine hepatocyte growth factor (HGF), and HGF signalling is often simultaneously activated in hypoxic conditions. Koga et al. reported that HGF interacted with Met, the receptor of HGF, to enhance the synthesis of HIF-1α via the MAPK and PI3K pathways in T24 cells. These up-regulation pathways may be attributed to the activation of VEGF, leading to the its binding with VEGFR [34]. Heat shock protein 90 is essential in HIF-1α folding and maturation. The heat shock protein 90 inhibitor geldanamycin substantially decreased hypoxia-induced VEGF expression in T24 cells [34]. Xia et al. discovered that pyruvate kinase M2 bound with STAT3 to form a protein complex, which was subsequently translocated into the nucleus where it stimulated HIF activity to induce angiogenesis in an oncogenic HRas-driven urothelial tumor mouse model [35].

Currently, targeted therapy for bladder cancer has shown limited efficacy. However, HGF, HSP90, pyruvate kinase M2, and STAT3 all hold potential as therapeutic targets for bladder cancer, as they play a role in anti-angiogenesis.

### Angiogenesis in PCa

In PCa, the degradation of HIF is reduced under hypoxic conditions, and cytokines and growth factors regulate HIF levels through mitogen-activated protein kinase (MAPK) or phosphatidylinositol 3-kinase (PI3K) pathways. By activating PI3K, the mammalian target of rapamycin (mTOR) phosphorylates p70 S6 kinase (p70S6K). The ribosomal S6 protein undergoes phosphorylation by p70S6K, which triggers HIF-1α synthesis. Triggering the MAPK pathway, extracellular signal-regulated kinase also phosphorylates p70S6K, as well as eukaryotic translation initiation factor 4E-binding protein 1. Phosphorylated 4E-binding protein 1 fails to combine with eukaryotic translation initiation factor 4E and induces the translation of HIF. PTEN, a tumor suppressor gene and is often mutated in PCa [36]. Fang et al. suggested that as a PI3K inhibitor, PTEN restrained the expression of both HIF and VEGF to inhibit angiogenesis in PCa [37]. Ci et al. discovered that transcription factor Krüppel-like factor 5 inhibited PI3K/AKT activity to reduce the rate of HIF and VEGF synthesis [38]. Sphingosine kinase 1 (SphK1) was stimulated in low oxygen conditions and activated Akt/glycogen synthase kinase-3B to prevent VHL-mediated HIF degradation [39, 40]. Then, SphK1 induced the accumulation of VEGF to promote angiogenesis in PCa. Insulin has been confirmed to enhance the creation of reactive oxygen species, such as H_2_O_2_, to activate p70S6K, leading to the upregulation of HIF and VEGF. Catalase has been demonstrated to abolish insulin-induced p70S6K activation [41]. Prostaglandin E2 (PGE2) is transported into cells by prostaglandin uptake transporter. Intracellular PGE2 interacts with intracellular EP2 receptors to exert its proangiogenic effect through the increased expression of HIF-1α [42]. It has been confirmed that HIF could induce the transition from respiratory metabolism to glycolytic metabolism, and an increased glycolytic rate promoted lactate production [43]. Luo et al. indicated that lactic acid produced in a hypoxic tumor environment was transported into cells by Monocarboxylate Transporter 1, and lactate inhibited HIF degradation in a hypoxia-independent manner [44]. In the process of angiogenesis, tumor microenvironment can be divided into hypoxic regions and normoxic regions. Lactate produced in hypoxic regions is transported to normoxic regions through blood vessels and could be absorbed by surrounding cells [45]. Intracellular lactate promotes HIF lactylation to stabilize HIF-1α, which enhances the transcription of KIAA1199. KIAA1199 has been found to depolymerize hyaluronic acid and initiate angiogenesis by stimulating VEGFA and inhibiting semaphoring 3 A action [44].

Current targeted therapies for prostate cancer appear to have limited effectiveness. The VEGF-A monoclonal antibody, Bevacizumab, has failed to provide PSA relief in CRPC as a monotherapy and has not achieved the survival endpoint when used in combination with other drugs for metastatic CRPC. Moreover, drugs targeting VEGF and its receptors have been shown to develop resistance after achieving certain therapeutic effects, indicating the need for novel targets such as HIF, KIAA1199, and metabolic pathways, which should be given due attention.

## HIF and Lymphangiogenesis

During embryonic development, lymphangiogenesis occurs later than angiogenesis, and VEGF-C, VEGF-D, as well as VEGFR-3 pathways dominate lymphangiogenesis [46, 47]. Under hypoxic conditions, both VEGF-C and VEGF-D are regulated by HIF. A vasodilator-vasoconstrictor imbalance triggered by hypoxia leads to malignant behavior of lymphatic endothelial cells (LECs). Nitric oxide is a common vasodilator, and the inhibition of itric oxide synthesis can block lymphatic hyperplasia [48]. Endothelin-1, a vasoconstrictor, is upregulated under hypoxic conditions by the transactivation of activator-protein-1 in LECs. Endothelin-1 binds to the endothelin B receptor located on LEC surfaces to induce HIF-1α expression and causes lymphatic cell proliferation mediated through the MAPK and PI3K pathways, leading to vessel branching [49]. In addition, hypoxic conditions increase the expression of Prospero-related homeobox1 (PROX1), which controls the key transformation of blood vascular endothelial cells into LECs in the embryonic period and, in turn, binds to the promoter of VEGFR-3 to enhance the expression of VEGFR-3 on LEC membrane [50, 51].

Hypoxia modulates macrophage action in both inflamed areas and malignant tumors. Macrophages in the circulation system are recruited to tumor microenvironment and located in the low-oxygen region [52]. HIF-1α links macrophages with this hypoxic environment, regulating its recruitment. Macrophages in hypoxic regions display abnormal metabolic patterns and overexpress HIF-1α and VEGF [48]. Macrophages secrete several cytokines, such as interleukin-1β (IL-1β), Migration-inhibitory factor (MIF), and VEGF-C/D in hypoxic conditions. MIF expressed by macrophages is stimulated under hypoxic conditions, and through the interactions with CD74, resulting in the accumulation of HIF-1α [53]. IL-1β modulates HIF-1α upregulation and activates target gene VEGF transcription through nuclear factor kappa B pathway [54]. IL-1β triggered VEGF-C/-D release in macrophages to promote lymphangiogenesis in mouse airways [55]. Tumor-associated macrophages respond to hypoxia to induce HIF-1α, which results in lymphangiogenesis.

Cyclooxygenase-2 is highly expressed in tumor cells. Its synthesized product, prostaglandin E2 binds to the EP1 receptor to promote the expression of VEGF-C, thereby facilitating lymphangiogenesis [56]. Additionally, VEGF-D inhibits 15-PDGH to block the degradation of prostaglandins to induce lymphatic vessel hyperplasia, which is engaged in tumor lymphatic metastasis [57]. The induction of lymphangiogenesis by HIF-1α is not mediated solely through VEGF signaling. Hypoxia increases chemokine CXCL12/CXCR4 transcription, and HIF-1α mediates CXCR4 production via VEGF-C in LECs. CXCL12 promotes lymphangiogenesis independent of VEGF-C/-D pathways [58].

Lymphangiogenesis is commonly considered as a mechanism that promotes tumor metastasis. VEGF-C stimulates the formation of new lymphatic vessels, allowing tumor cells to bypass lymph nodes and rapidly spread to distant organs [59]. In RCC, the impact of VEGF-C on survival is somewhat contradictory. In patients without metastasis and with lower histological grades of the tumor, higher levels of VEGF-C are associated with better prognosis, manifested by longer DFS and OS. However, in metastatic patients, higher expression of VEGF-C is associated with poorer prognosis [60].

This duality may be attributed to the inherent characteristics of the lymphatic system. During the early stages of RCC development, generated lymphatic vessels drain tumor cells to the lymph nodes, allowing immune cells to recognize the tumor cells more quickly and trigger an anti-tumor immune response. From this perspective, VEGF-C plays an anti-tumor role in the initial stages of tumor development, leading to improved patient survival. However, when tumor cells invade the lymph nodes, VEGF-C-induced lymphatic vessel formation accelerates tumor metastasis and recruits suppressive immune cells, impairing the anti-tumor immune response [61].

**Fig. 1:**
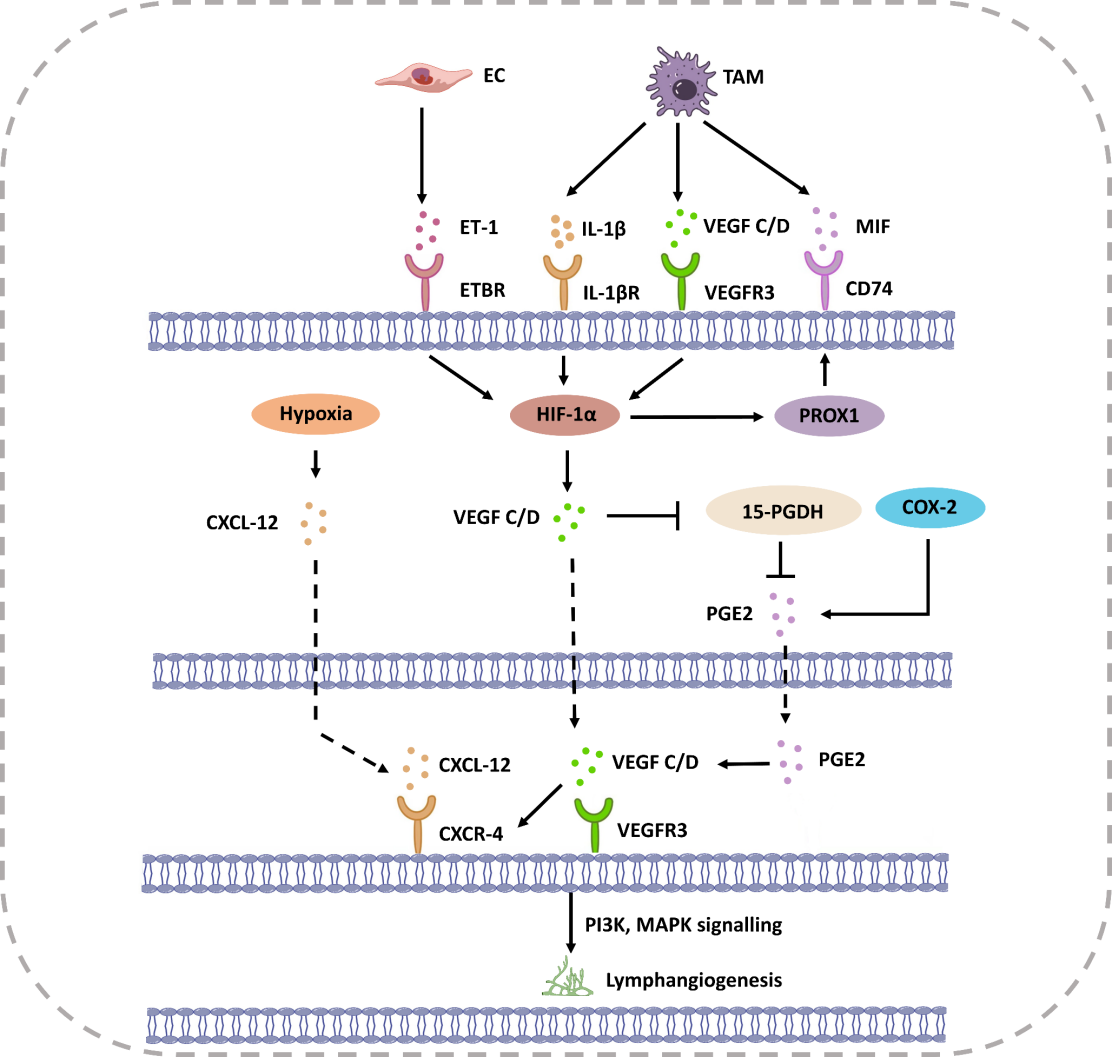
Regulation of tumor-associated lymphangiogenesis by HIF-1α

In RCC, the regulation of VEGF-C by HIFs can vary. HIF-1 binds to the promoter of VEGF-C in normal kidney tissue, promoting transcription and increasing lymphatic vessel formation [62]. As mentioned earlier, most RCCs have high expression of HIF-2. Ndiaye et al. demonstrated that HIF-2 promoted the production of VEGF-C protein but inhibited its transcription in VHL-inactivated RCC cell lines [60]. Previous studies have shown that hypoxia can reduce the VEGF-C mRNA but enhance protein expression of VEGF-C by affecting intra-ribosomal entry sites within its 5’UTR [63]. Additionally, the activity of VEGF-C’s promoter is mainly influenced by the binding strength of NFκB. Hypoxia-induced dephosphorylation of NFκB can interfere with its binding to the promoter, thereby inhibiting transcription and potentially causing a decrease in mRNA levels [60]. The regulatory patterns described above suggest that VEGF-C may be influenced by different mechanisms at the mRNA and protein levels, necessitating further experiments to elucidate the regulatory relationship between them Fig. 1.

## HIF and Tumor Microenvironment

The characteristics of tumor microenvironment are complexity and continuous variability. It comprises different types of cells, including immune cells, blood vessels, stromal cells, and lymphatic vessels. The communication between malignant cells and other components in the TME promotes the malignancy of cancers. With the rapid growth of cancer cells, nutrients are inadequately provided by the existing vessel systems. Hypoxia and increased acidity are typical characteristics of tumor microenvironment. To overcome these challenges, TME cells secrete proangiogenic factors to facilitate angiogenesis and lymphangiogenesis, resulting in the significant infiltration of lymphocytes. These molecules and cells exert negative effects on antitumor immunity, which may eventually lead to an immunosuppressive microenvironment [64].

Hypoxia within the TME can lead to immune suppression. HIF-1α signalling mediates PD-L1 expression in several immune-suppressive cells [65]. Macrophages in hypoxic regions of tumors are polarized into the M2-like phenotype [66]. The hypoxic TME also recruits regulatory T cells (Tregs) via the secretion of CC-chemokine ligand 28 [67].

Hypoxia-induced HIF-1α accumulation activates fibroblast through the Sonic Hedgehog (Shh) pathway. Connective tissue growth factor and transforming growth factor-β of fibroblasts are upregulated by HIF-1α [68]. Activated fibroblasts undergo contraction, proliferation, and overproduction of extracellular matrix (ECM) components to promote desmoplasia and fibrosis [68]. In addition, hypoxia causes the generation of blood and lymphatic vessels. Thus, fibroblasts and excessive ECM cause physical stress and compress the newly formed vessels. The compressed blood vessels exhibit impaired blood perfusion, which may aggravate hypoxia in the TME [69]. Constricted lymphatic vessels also show impaired drainage, leading to elevated interstitial fluid pressure, which further reduces blood flow [48, 70]. Hypoxia eventually compromises antitumor immunity. Cancer-associated fibroblasts (CAFs) directly induce immunosuppression. Feig et al. demonstrated that FAP + CAFs secreted CXCL12 and inhibited T lymphocyte activities [71]. CAFs also impaired NK cell cytotoxic activities by disrupting cell receptor expression and reducing cytokine production [68, 72].

The neovascularization of tumors often leads to structural anomalies and vessel leakiness. Endothelial cells display irregularities and polarity lost, detaching from the vascular wall and stacked together. Pericytes detach from ECs and lose their capacity to regulate blood flow and permeability [73]. The basement membrane of newly formed vessels forms pores and shows uneven thickness, compromising the normal positioning of endothelial cells and pericytes [74]. The loss of tumor vascular integrity results in vessel leakiness. Additionally, the newly formed lymphatic vessels may lack functionality, and hyperplasia of these vessels can result in insufficient valve closure, leading to drainage obstruction [48]. Blood vessel leakage and obstructed lymphatic drainage exacerbate hypoxia within the TME.

**Fig. 2:**
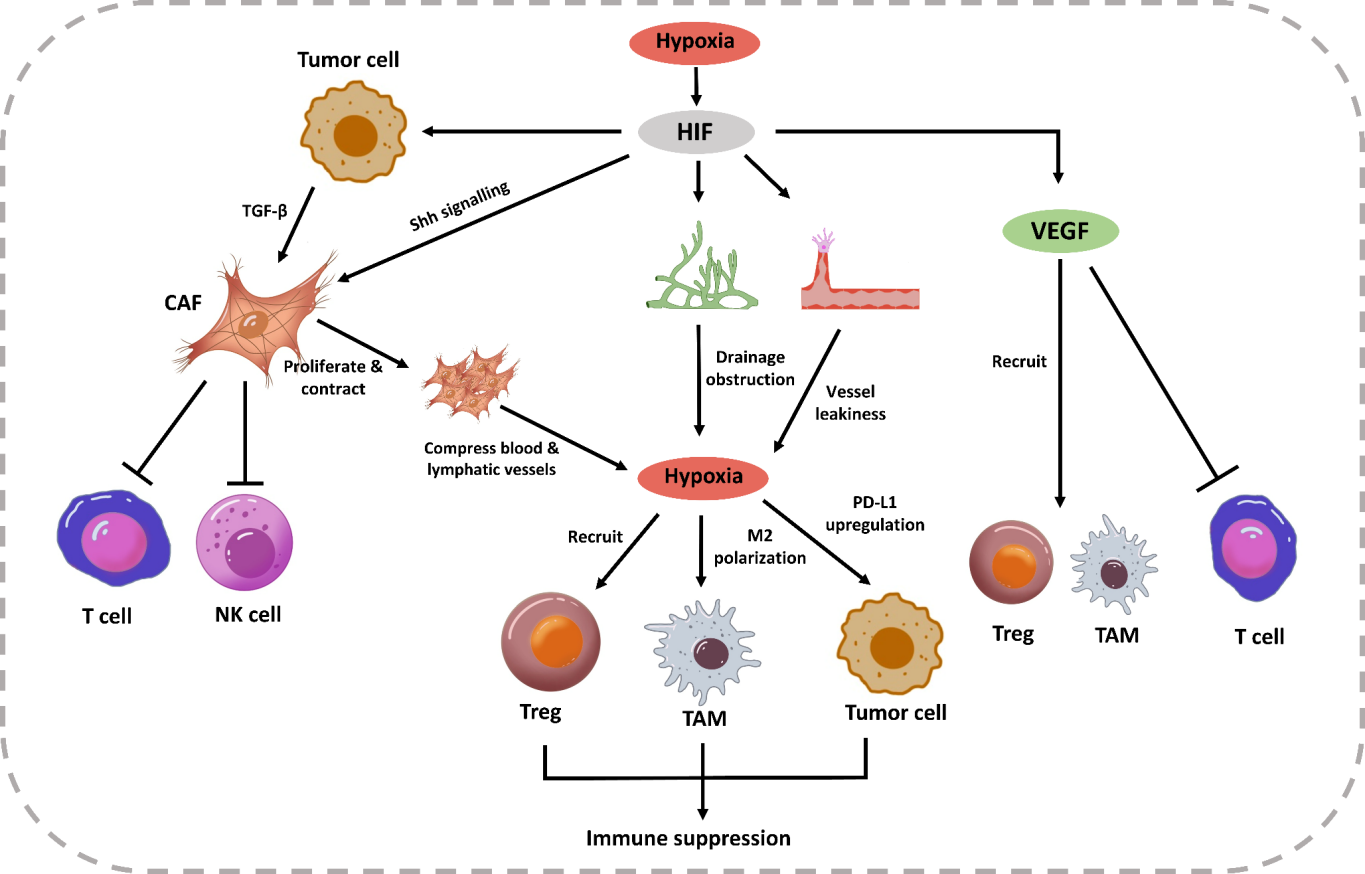
Schematic overview of HIF-induced angiogenesis, lymphangiogenesis and immune suppression

The VEGF signalling pathway facilitates immune suppression through interactions with other stromal elements. VEGF binds to VEGFR2 on T cells to increase the expression of immune checkpoints. This process also significantly inhibits the cytotoxicity of effector T cells [75, 76]. VEGF inhibits monocyte differentiation into dendritic cells and their maturation, dampening dendritic cell antigen presentation [77]. VEGF recruits Tregs to tumors and promotes the polarization of macrophages into M2 tumor-associated macrophages (TAMs) [78, 79]. Hypoxia-induced angiogenesis has been positively correlated with TAM infiltration in BCa [16]. Kusmartsev et al. reported that VEGFR-1 enhanced the inhibitory capacity of myeloid-derived suppressor cells in RCC [80]. Wang et al. reported that the FOXA1/HIF-1α axis was connected with the epithelial-mesenchymal transition (EMT) and TAM infiltration in PCa [81] Fig. 2.

## Targeted therapy

As mentioned previously, HIF is crucially involved in the progression of tumors, and the upregulation of HIF-1α and HIF-2α has been proved to indicate an unfavorable prognosis in numerous cancer patients. Therefore, targeting HIF and its downstream signalling may be potential strategies in clinical therapy. The Food and Drug Administration has approved vorinostat, a histone deacetylase inhibitor, for treating cutaneous T-cell lymphoma. Hutt et al. stated that vorinostat inhibits the translation of HIF-1α and may be a potential approach in cancer treatment [82]. Belzutifan (MK-6482) is a HIF-2α inhibitor and has led to a favourable objective response rate and relatively mild adverse effects in patients with nonmetastatic RCC [83].

Although inhibition of lymphangiogenesis is considered a potential strategy target for cancer treatment, only a few drugs have been applied specifically to inhibit this process thus far. Lymphangiogenesis is triggered mainly by VEGF-C and VEGF-D pathways. IMC‑3C5 is an anti-VEGFR-3 monoclonal antibody that is under evaluation in a Phase I clinical trial with colorectal cancer patients. Although the drug showed good tolerability, its antitumor efficacy was limited [84]. VGX-100, a VEGF-C-blocking antibody, is currently evaluated in an advanced solid tumor clinical trial for which the results have not yet been published (ClinicalTrials.gov number, NCT01514123).

Targeted therapy often involves targeting the VEGF signalling pathway to suppress angiogenesis. Although long-term antiangiogenic therapy may exacerbate tumor hypoxia, recent research has indicated that after a period of antiangiogenic drug use, tumor vasculature was normalized, which increased tumor cell sensitivity to immunotherapy or chemotherapy [24, 68]. Therefore, antiangiogenesis-targeted therapy is often combined with immune checkpoint inhibitors in the therapy of metastatic ccRCC. Cabozantinib plus nivolumab, axitinib plus pembrolizumab, and lenvatinib plus pembrolizumab have become standards of care for metastatic ccRCC, according to European Association of Urology recommendations. Other combination therapies are currently in clinical trials, which are listed in Table 1.

**Table 1:** Overview of drugs in clinical testing which target angiogenesis pathways and drug combinations for systemic therapy

Drug name	Target	Cancer Type	Current status	NCT number
Sunitinib + Abemaciclib	VEGFR-1/2/3 + CDK4/6	mRCC	Phase I	NCT03905889
XL092 + Nivolumab	VEGFR-2 + PD-1	RCC	Phase III	NCT05678673
Axitinib + Toripalimab	VEGFR-1/2/3 + PD-1	mRCC	Phase III	NCT04394975
Bevacizumab + Pazopanib	VEGF-A + VEGFR-1/2/3	mRCC	Phase I/II	NCT01684397
Pazopanib + Bevacizumab + Everolimusa	VEGFR-1/2/3 + VEGF-A + mTOR	mRCC	Phase II	NCT01217931
Sunitinib + Temsirolimus	VEGFR-1/2/3 + mTOR	mRCC	Phase II	NCT01517243
Axitinib + Avelumab	VEGFR-1/2/3 + PD-L1	mRCC	Phase III	NCT02684006
Anlotinib + TQB2450	VEGFR-1/2/3 + PD-L1	mRCC	Phase III	NCT04523272
Lenvatinib + Envafolimab	VEGFR-1/2/3 + PD-L1	mRCC	Phase I/II	NCT05024214
Bevacizumab + TRK-950	VEGF-A + CAPRIN-1	mRCC	Phase I	NCT03872947
Trebananib + Pembrolizumab	Ang-2 + PD-1	mRCC	Phase I	NCT03239145
ESK981	VEGFR-1/2 + Tie-2	mCRPC	Phase II	NCT03456804
Sunitinib + Abiraterone + Prednisone	VEGFR-1/2/3 + CYP17A1	mPCa	Phase II	NCT01254864
Sunitinib + Leuprolide	VEGFR + LH, FSH	localized PCa	Phase II	NCT00329043
cabozantinib + atezolizumab	VEGFR-1/2/3 + PD-L1	CRPC, RCC and BCa	Phase I/II	NCT03170960
Resveratrol	VEGF + FGF-2	BCa	Preclinical	-
Carnosine	VEGFR-2	BCa	Preclinical	-
EV-CTSB	VEGF-A	BCa	Preclinical	-

Abbreviations: VEGFR, vascular endothelial growth factor receptor; CDK, cyclin-dependent kinase; mRCC, metastatic renal cell carcinoma; PD-1, programmed cell death protein 1; RCC, renal cell carcinoma; VEGF, vascular endothelial growth factor; PD-L1, programmed death-ligand 1; mTOR, mammalian target of rapamycin; CAPRIN-1, cytoplasmic activation/proliferation-associated protein-1; Ang-2, angiopoietin-2; Tie-2, tyrosine kinase with immunoglobulin-like and EGF-like domains 2; mCRPC, metastatic castration-resistant prostate cancer; CYP17A1, cytochrome p450 17a1; mPCa, metastatic prostate cancer; LH, luteinizing hormone; FSH, follicle-stimulating hormone; PCa, prostate cancer; BCa, bladder cancer; FGF2, fibroblast growth factor 2.

Antiangiogenic monotherapy has led to relatively few clinical benefits in metastatic BCa [85]. A recent Phase III trial assessed the effectiveness of adding bevacizumab to gemcitabine and cisplatin in advanced BCa patients, and no improvement in overall survival (OS) was observed [86]. Further exploration and clinical evidence are required to determine the potency of antiangiogenic therapy and other combination therapies in BCa.

Although antiangiogenic therapy has shown limited efficacy in PCa, new targeted monotherapies or combination therapies are under development. ESK981 (CEP-11,981) is a multityrosine kinase inhibitor targeting VEGFR-1/2 and Tie-2 and is in a Phase II clinical trial to evaluate its potency in treating CRPC [87]. Atezolizumab plus cabozantinib has been entered into a Phase II clinical trial for treating CRPC patients whose tumors have progressed in the soft tissue during or after new androgen pathway targeting agents treatment for metastatic disease.

## Discussion

The regulation of HIFs in response to hypoxia is a crucial process in tumorigenesis. Among the HIF family, HIF-1 and HIF-2 are the most extensively studied molecules, and they are often found to act as oncogenes in many types of tumors, including bladder cancer and prostate cancer. However, in ccRCC, HIF-1 exhibits tumor-suppressive effects, while HIF-2 promotes tumor growth. Interestingly, a study conducted by Hoefflin et al. revealed that HIF-1 is essential for the initiation formation of ccRCC, suggesting its potential role in carcinogenesis [31]. These seemingly contradictory findings can be attributed to a couple of factors. Firstly, the study was performed using a mouse model, which may not fully replicate the TME in human beings. Secondly, HIF-1 and HIF-2 may exert their effects at different periods of tumor development, indicating a dynamic balance between them. The inactivation of VHL leads to the elevation of HIF-1/2 in precancerous lesions. Increased levels of HIF-1 activate tumor formation, while elevated levels of HIF-2 result in suppression of HIF-1.

In bladder cancer and prostate cancer, the majority of research on angiogenesis has focused on the impact of HIF-1 on VEGF and its receptors. However, the role of HIF-2 and HIF-3 in these cancers remains largely unexplored. It appears that HIF-1 and HIF-2 work in synergy to promote tumor development, but the dynamic regulation between them, as observed in renal cancer, still requires further experimental exploration.

HIFs are multifunctional transcription factors that can target multiple downstream pathways to promote angiogenesis. VEGF and angiopoietin 2 bind to receptors located on the surface of endothelial cells, while stromal-derived factor 1 and stem cell factor bind to receptors on bone marrow cells to facilitate vascularization. In urologic tumors, angiogenesis is primarily associated with the VEGF pathway, neglecting the contribution of these targets to angiogenesis. However, focusing on these targets collectively may lead to discoveries in anti-angiogenic therapy.

Lymphangiogenesis in urologic tumors has not been thoroughly investigated, and existing studies primarily focus on the major regulatory factors VEGF-C/D and their interplay in tumor development. In human breast cancer tissue, the localization of HIF-1 and PDGF-B is proximal. PDGF-B has been found to promote immigration and growth of lymphatic endothelial cells in a mouse fibrosarcoma cell line [88], and in breast cancer cell lines, HIF-1 can enhance lymphangiogenesis by upregulating PDGF-B, indicating the significant influence of PDGF-B in lymphatic vessel formation [89]. Further mechanistic research and clinical trials are needed to validate its role in genitourinary tumors.

The presence of VHL mutations in RCC directly leads to elevated expression of HIFs and anti-angiogenic therapy has achieved encouraging results in the treatment of RCC. Targeted therapy and immunotherapy have been extensively employed in the management of metastatic RCC with ongoing clinical trials aiming to explore the most effective drug combinations.

The response to immunotherapy is determined by different immune microenvironments.

Bladder cancer, characterized by a high tumor mutation burden, demonstrates a favorable response to immunotherapy. Nivolumab has achieved notable efficacy in the DFS of muscle-invasive bladder cancer [90]. However, when it comes to monotherapy with anti-angiogenic agents, no benefits have been observed. Prostate cancer patients have limited benefits from immunotherapy targeting PD-1/PD-L1. This is related to the low tumor mutation burden, limited generation of neoantigens, and the presence of immunosuppressive immune cells in prostate cancer. On the other hand, anti-angiogenic therapy plays a crucial role in restoring abnormal tumor vasculature to a normal functional state. It could enhance the sensitivity to immunotherapy. However, VEGF blockade encounters resistance in prostate cancer. These challenges call for new targets that may bring about a breakthrough in targeted therapy and immunotherapy for prostate cancer.

In conclusion, we present a thorough explanation of how HIFs serve as critical transcription factors in driving tumorigenesis, angiogenesis, and lymphangiogenesis, and describe their interaction with stromal elements of tumor microenvironment. Furthermore, we discuss the application of targeted therapies against HIFs and downstream pathways, as well as their combination therapies in urological cancers. Our goal is to establish a foundation for future studies on hypoxia-related molecular mechanisms and identify new therapeutic targets for urological tumors.

## Data Availability

Not applicable.

## Abbreviations

BCa: Bladder cancer
CAF: cancer-associated fibroblast
CRPC: castration-resistant PCa
chRCC: chromophobe RCC
EC: endothelial cell
EMT: epithelial-mesenchymal transition
ECM: extracellular matrix
HGF: hepatocyte growth factor
HIF: hypoxia-inducible factor
IL-1β: interleukin-1β
LEC: lymphatic endothelial cell
mTOR: mammalian target of rapamycin
MVD: microvessel density
MAPK: mitogen-activated protein kinase
OS: overall survival
pRCC: papillary RCC
PI3K: phosphatidylinositol 3-kinase
p70S6K: phosphorylates p70 S6 kinase
PDGF: platelet-derived growth factor
PROX1: prospero-related homeobox1
PGE2: prostaglandin E2
PCa: prostate cancer
Treg: regulatory T cell
RCC: renal cell carcinoma
SNP: single nucleotide polymorphism
Shh: sonic hedgehog
SphK1: sphingosine kinase 1
TME: tumor microenvironment
TAM: tumor-associated macrophage
VEGF: vascular endothelial growth factor
pVHL: VHL gene product
VHL: Von Hippel–Lindau
